# Sub-wavelength visualization of near-field scattering mode of plasmonic nano-cavity in the far-field

**DOI:** 10.1515/nanoph-2022-0679

**Published:** 2023-01-16

**Authors:** Xiao Jin, Shengwei Ye, Weiqing Cheng, Jamie Jiangmin Hou, Wanzhen Jin, Tianyao Sheng, Lianping Hou, John H. Marsh, Yefeng Yu, Ming Sun, Bin Ni, Xuefeng Liu, Jichuan Xiong

**Affiliations:** School of Electronic and Optical Engineering, Nanjing University of Science and Technology, Nanjing 210094, P. R. China; James Watt School of Engineering, University of Glasgow, Glasgow, G12 8QQ, UK; Department of Medicine, University of Cambridge, Hills Road, Cambridge, CB2 0QQ, UK

**Keywords:** diffraction limit, far-field, PIMI, plasmonic mode detection

## Abstract

Spatial visualization of mode distribution of light scattering from plasmonic nanostructures is of vital importance for understanding the scattering mechanism and applications based on these plasmonic nanostructures. A long unanswered question in how the spatial information of scattered light from a single plasmonic nanostructure can be recovered in the far-field, under the constraints of the diffraction limit of the detection or imaging optical system. In this paper, we reported a theoretical model on retrieving local spatial information of scattered light by plasmonic nanostructures in a far-field optical imaging system. In the far-field parametric sin *δ* images, singularity points corresponding to near-field hot spots of the edge mode and the gap mode were resolved for gold ring and split rings with subwavelength diameters and feature sizes. The experimental results were verified with Finite Difference Time Domain (FDTD) simulation in the near-field and far-field, for the edge mode and the gap mode at 566 nm and 534 nm, respectively. In sin *δ* image of split-ring, two singularity points associated with near-field hot spots were visualized and resolved with the characteristic size of 90 and 100 nm, which is far below the diffraction limit. The reported results indicate the feasibility of characterizing the spatial distribution of scattering light in the far-field and with sub-wavelength resolution for single plasmonic nanostructures with sub-wavelength feature sizes.

## 1 Introduction

Localized Surface Plasmon Resonance (LSPR) is a resonance phenomenon of conduction electrons generated at the metal surface [1]. Recently, based on near-field enhancement, i.e., hot spots of LSPR at resonance wavelengths, plasmonic nanostructures are being widely used in fields such as Surface-Enhanced Fluorescence (SEF) [2, 3]. Surface-Enhanced Raman Scattering (SERS) [4, 5], metamaterial [6, 7], nonlinear optics [8, 9], photothermal conversion [10, 11], and plasmonic laser [12, 13].

Observation of optical excitation and interaction of specific plasmonic modes in metal nanostructures is an important experimental process for the design and application of these nanostructures. However, due to the far-field diffraction limit, a spatial mode distribution can hardly be visualized for subwavelength plasmonic structures, most research studies focus on characterizing their spectral phenomena [14–16]. In some research studies, plasmonic modes are clarified as polarization polar image under specific wavelength, which is still formed based on spectral measurements and spatial information of the resonance modes remain uncovered [17–19].

To achieve far-field spatial imaging of plasmonic modes, fluorescent dyes are used as labels to indicate spatial distribution and localized hot spots of plasmonic emission [20]. However, this method relies on the stochastic super-resolution microscopy which involves switching fluorescent molecules and image reconstructions after multiple sequenced measurements.

Compared to far-field methods, near-field microscopes are more frequently used for characterizing the near-field spatial modes with a high resolution down to the nanometer level. Photo-induced Force Microscopy (PiFM) [21, 22], Photon Scanning Tunneling Microscope (PSTM) [23, 24] and Scanning Near-field Optical Microscopy (SNOM) [25–27] are typical optical methods which detect near-field electric field directly. As optical systems, they can naturally be applied to complicated plasmonic samples with biological components. Another kind of representative near-field microscope is Photoemission Electron Microscopy (PEEM) [28, 29]. Under the photon-electric effect, its spatial resolution does not depend on the incident electromagnetic light field, but on the electron detection optics. These microscopes could all reach a resolution of around 10 nm. However, regardless of their expensive and time-consuming operation and requirement for vacuum environment, they have a common problem: parasitic coupling effects between near-field probe and object often hinder even qualitative signal interpretation [25]. So, a spatial resolving method for characterizing plasmonic modes distribution in far-field is still in high demand.

In this article, we provide a far-field mode distribution imaging system for characterizing subwavelength plasmonic nanostructures. Based on an indirect polarization imaging technique (PIMI, polarized indirect microscopic imaging) [30, 31], the system transfer near-field polarization information of the scattered light into far-field parametric, i.e., sin *δ*, images with deep sub-wavelength resolving capability.

From the theoretical calculations, the measured indirect parameters can be related to the polarization difference between the transverse and longitudinal modes which contain mode distribution and singularity information. Due to the fact that the denominator of one term in equations representing the contrast of the detected signal will turn to zero under some conditions, singularity points with high gradience could be achieved at certain imaging locations. In other words, the characteristic size of singularity points far below the diffraction limit could be generated for the corresponding pixels in the imaging results. Also, this method shows high robustness to wavelength change, and mode characters can be stably resolved at wavelength far away from the resonant peak.

To verify the theoretical model, plasmonic ring and split-ring nanodots arrays are fabricated and different modes were measured. With assistance of Finite Difference Time Domain method (FDTD), near-field electromagnetic field is simulated and related to the imaged far-field mode distribution.

An edge mode at 566 nm wavelength is detected both in ring and split-ring structure, which is accompanied with hot spots on its outer surface. Another resonant peak was found at 534 nm, corresponding to a gap mode in transverse direction for a split-ring. The field in the cavity gap is strongly enhanced in this mode. In the PIMI sin *δ* images of the split-ring structure, gap modes combined with edge modes lead to two singularity points in the longitudinal orientation, which is associated with the split-ring size. In addition, our method can show greater details under wavelength far away from resonant peak, and possess high robustness. Similar to other indirect imaging methods like quantitative phase gradient microscopy [32] or differential phase-contrast microscopy [33, 34], the imaging ability could be analyzed from features in experimental results. The characteristic size of two singularity points could be as small as 90 nm, much smaller than the diffraction limit, and suggests an efficient far-field high resolution microscopic method for plasmonic structure modes visualization with a relatively large field of view.

## 2 Theoretical framework

The optical system is based on the PIMI method, which is utilized to measure the polarization ellipse orientation angle and phase difference between two orthogonal directions of light transmitted, reflected or scattered from samples.

In this system, a rotating polarizer is put in front of the illumination source with a rotating angle *θ*_*i*_ and reflected by a semi-transparent mirror. After focusing by an objective, the reflected and scattered light will pass through a quarter wave plate and a polarizer with an angle of 45° (more details in Supporting Information and Figure S1). The formula of the output light field is

(1) $$I_{i}=\frac{1}{2}I_{0}\left[1+\sin\delta \;\sin2\left(\theta_{i}-\phi \right)\right]$$

Here *I*_*i*_ (the subscript *i* indicates the number of polarization rotation angles) is the pixel intensity in each measured image. *I*_0_ represents the average intensity under all illumination polarization states, which is also equivalent to the intensity obtained by a conventional microscope. Sin *δ* is the sine of the phase difference between two orthogonal polarization components of the scattered light. *θ*_*i*_ is the polarization angle of the linearly polarized incident beam and *ϕ* is the polarization ellipse orientation angle of the beam reflected from the sample.

The same system could be utilized for subwavelength nanostructures, to reveal the subwavelength mode distribution of plasmonic structure in PIMI results, the image contrast from the conventional microscope and PIMI system is theoretically compared as below.

We assume two kinds of modes could be excited in an axial symmetric plasmonic nanostructure under unpolarized illumination. When illuminated by polarized light under the *x*-axis, the 2D far-field mode distribution is named the transverse mode $\vec{E_{t}}\left(r\right)$ . The mode under polarized light in the *y*-axis is the longitudinal mode $\vec{E_{l}}\left(r\right)$ . Here ***r*** = (*x*, *y*) represents the position in the *x*–*y* plane.

Considering that electrons in plasmonic structures oscillate with the polarization direction of the incident electric field, most of the energy for transverse and longitudinal modes will concentrate on the *x* and *y* components of electric fields, respectively. Especially for symmetric structures, vertical components could theoretically reduce to zero along the *x* and *y* axes.

$$\vec{E_{t}}\left(r\right)=\left(E_{t}\left(x,y\right),0,0\right)$$

(2) $$\vec{E_{l}}\left(r\right)=\left(0,E_{l}\left(x,y\right),0\right)$$

When the polarizer in PIMI system rotates at an angle *θ*_*i*_, the output far-field electric field ${\vec{E}}_{i}\left(x,y\right)$ should be a linear combination of transverse and longitudinal modes.

(3) $${\vec{E}}_{i}\left(x,y\right)=\left(\cos\theta_{i}\cdot E_{t}\left(x,y\right),\sin\theta_{i}\cdot E_{l}\left(x,y\right)\right)$$

Thus, the intensity distribution should be

(4) $$\begin{array}{ll} I_{i}\left(x,y\right) & =cos^{2}\theta_{i}\cdot E_{t}{\left(x,y\right)}^{2}+sin^{2}\theta_{i}\cdot E_{l}{\left(x,y\right)}^{2}\\ & =E_{t}{\left(x,y\right)}^{2}+E_{l}{\left(x,y\right)}^{2}+\left(E_{t}{\left(x,y\right)}^{2}-E_{l}{\left(x,y\right)}^{2}\right)\\ & \;\times cos2\theta_{i} \end{array}$$

It is straightforward to derive that

(5) $$\sin\delta =\frac{E_{t}{\left(x,y\right)}^{2}-E_{l}{\left(x,y\right)}^{2}}{E_{t}{\left(x,y\right)}^{2}+E_{l}{\left(x,y\right)}^{2}}$$

Meanwhile the image from the traditional microscope is

(6) $$I_{0}=E_{t}{\left(x,y\right)}^{2}+E_{l}{\left(x,y\right)}^{2}$$

Now we can compare the contrast between sin *δ* and *I*_0_ from spatial differential values with respect to its original values at an arbitrary position. We can choose a point with infinitesimal displacement (Δ*x*, Δ*y*), which causes infinitesimal value change $\Delta {E_{t}}^{2}$ and $\Delta {E_{l}}^{2}$ . Then we get

(7) $$\begin{array}{ll} \frac{\Delta \sin\delta }{\sin\delta } & =\frac{\frac{E_{t}{\left(x,y\right)}^{2}+\Delta {E_{t}}^{2}-\left(E_{l}{\left(x,y\right)}^{2}+\Delta {E_{l}}^{2}\right)}{E_{t}{\left(x,y\right)}^{2}+\Delta {E_{t}}^{2}+E_{l}{\left(x,y\right)}^{2}+\Delta {E_{l}}^{2}}-\frac{E_{t}{\left(x,y\right)}^{2}-E_{l}{\left(x,y\right)}^{2}}{E_{t}{\left(x,y\right)}^{2}+E_{l}{\left(x,y\right)}^{2}}}{\left(\frac{E_{t}{\left(x,y\right)}^{2}-E_{l}{\left(x,y\right)}^{2}}{E_{t}{\left(x,y\right)}^{2}+E_{l}{\left(x,y\right)}^{2}}\right)}\\ & =\frac{\frac{\Delta {E_{t}}^{2}-\Delta {E_{l}}^{2}}{E_{t}{\left(x,y\right)}^{2}-E_{l}{\left(x,y\right)}^{2}}-\frac{\Delta {E_{t}}^{2}+\Delta {E_{l}}^{2}}{E_{t}{\left(x,y\right)}^{2}+E_{l}{\left(x,y\right)}^{2}}}{1+\frac{\Delta {E_{t}}^{2}+\Delta {E_{l}}^{2}}{E_{t}{\left(x,y\right)}^{2}+E_{l}{\left(x,y\right)}^{2}}} \end{array}$$

Ignoring the small quantity in the denominator, the expression will become

(8) $$\frac{\Delta \sin\delta }{\sin\delta }=\frac{\Delta {E_{t}}^{2}-\Delta {E_{l}}^{2}}{E_{t}{\left(x,y\right)}^{2}-E_{l}{\left(x,y\right)}^{2}}-\frac{\Delta {E_{t}}^{2}+\Delta {E_{l}}^{2}}{E_{t}{\left(x,y\right)}^{2}+E_{l}{\left(x,y\right)}^{2}}$$

Also, we have

(9) $$\frac{\Delta I_{0}}{I_{0}}=\frac{\Delta {E_{t}}^{2}+\Delta {E_{l}}^{2}}{E_{t}{\left(x,y\right)}^{2}+E_{l}{\left(x,y\right)}^{2}}$$

When observing the two formulas (8) and (9), the contrast of *I*_0_ is limited by mode intensity distribution itself. Due to the far-field resolution limit of diffraction, $\Delta {E_{t}}^{2}$ and $\Delta {E_{l}}^{2}$ will be much smaller than in near field, which result in a low sensitivity of image characters. However, except for a traditional term restricted by the diffraction limit, another term where the subtraction between the longitudinal mode and the transverse mode is generated in the denominator of expressing the contrast of sin *δ*, leading to possible infinity detectable signal values for two points with distance unconstrained by below the diffraction limit. Thus, PIMI sin *δ* images could restore affluent details about far-field mode spatial distribution when light intensity of point spread functions from two adjacent points could not be distinguished from results with conventional microscopes, and is only limited by detecting noise.

## 3 Sample preparation and system configuration

Our plasmonic nano structures are fabricated on the Si substrate. After spinning a layer of Polymethyl Methacrylate (PMMA) photoresist, the patterns are defined using electron beam lithography (EBL) and development. After that, a 10 nm thick titanium layer for stabilizing the connection and a 70 nm thick gold layer were deposited by employing the electron beam evaporation method (the complete processes are shown in Supporting Information).

**Figure 1:**
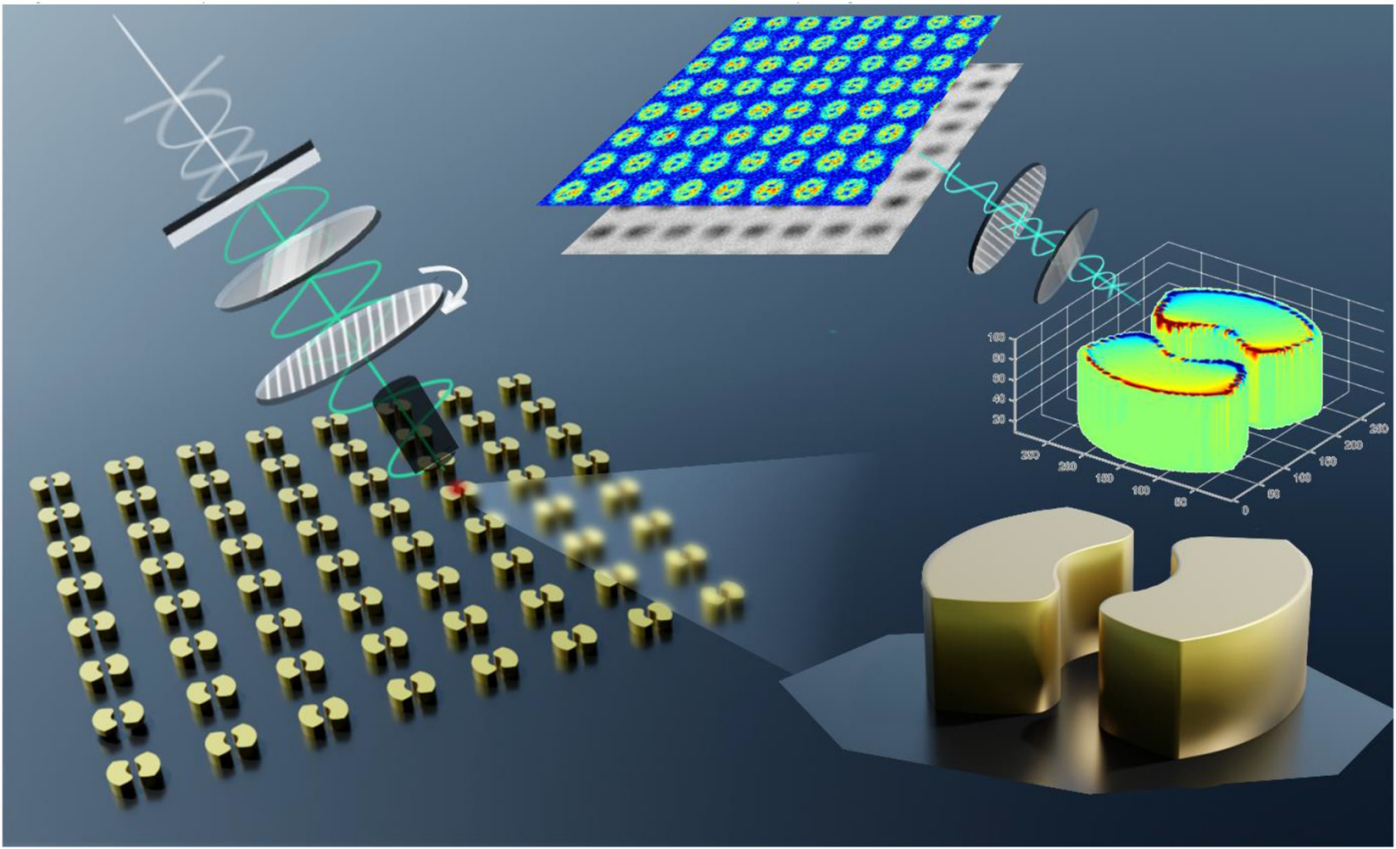
Schematic of plasmonic modes detection under indirect polarized imaging system.

A ring structure is designed with an outer diameter of 260 nm and an inner diameter of 80 nm. As shown in Figure 1, the split-ring structures share the same outer and inner diameters and a periodic interval of 1 μm is designed to avoid unnecessary coupling effects between two adjacent structures. Considering fabrication difficulty, a smooth curve is used to split the ring. More specifically, the curve possesses a ω-like shape and function of $y=0.0000015*{\left(x^{2}-3600\right)}^{2}+20$ , which also matches the size of the center hole used for the ring structure. The unit of formular is nanometer.

The experimental optical system is built based on a conventional reflection microscope (Olympus BX51). A liquid crystal tunable filter (Thorlab KURIOS-WB1) associated with a quarter-wave is placed in front of a halogen lamp source to select the illumination wavelength with a bandwidth of 35 nm. A series of images are recorded from a wavelength of 480 nm–680 nm with steps of 20 nm.

The process of PIMI measurement and row data are shown in Figures S1 and S2, where a polarizer is rotated by a motor from 0° to 180° in steps of 18°. The sampling number of rotating angles is set as 10, considering a balance between the imaging time and imaging noises (Figure S3 in Supporting Information). The light reflected by the sample was passed through a quarter-wave plate and a linear analyzer in front of the image plane with their fast axes oriented at 45° and 90° respectively relative to the *x*-axis. A highly sensitive CCD (PiA2400-17gm, Basler) with 5 million pixels was used in combination with a 100× objective lens with NA = 0.9, making it possible to obtain images with a pixel size of 34.5 nm. Average intensities for background in 18 row images are uniformed to reduce the system error. For analyzing with a more accurate position, linear interpolation is used to reshape image pixel size from 34.5 nm to 10 nm.

The spectrum results are acquired from a high-resolution spectrometer (Andor SR-500i), which reaches 0.03 nm in wavelength resolution. We used a dark-field configuration to reduce the impact of strong reflection from the silicon substrate. Only scattering lights are collected to analyze mode information in two plasmonic structures.

## 4 Results and discussions

**Figure 2:**
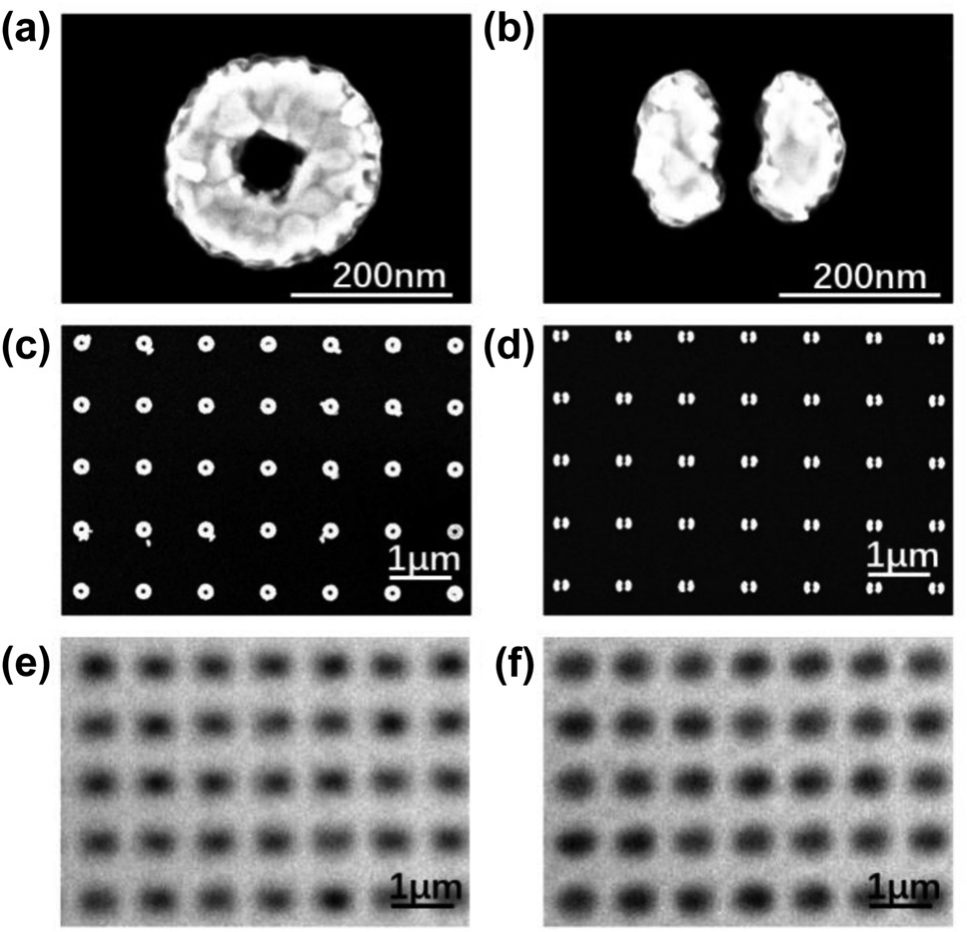
SEM images for ring and split-ring structures at (a)–(b) small scale and (c)–(d) large scale. Images for (e) ring and (f) split-ring structures from conventional microscope.

Scanning Electron Microscope (SEM) is used to verify the morphology of the fabricated structures. As shown in Figure 2(a) and (b), ring and split-ring structures are fabricated basically as designed. In Figure 2(c) and (d), images from a conventional microscope are indicated. Except for a minor difference in intensity, distribution for ring and split-ring both show similar blur spots at the position of every element in the array. Due to the diffraction limit of resolution, we cannot obtain enough details, especially the spatial distribution about modes and to distinguish these two structures.

As a comparison, a simulation based on FDTD is conducted to acquire near-field details about these two structures. We set a Total-Field Scattered-Field (TFST) source covering the structures with a wavelength range from 400 nm to 700 nm. Boundaries on all orientations are chosen as Perfect Matched Layer (PML).

To gain the resonance information for the two structures, we set a downward polarized plane wave vertical to Si substrate. For ring structure, polarization direction is set as the *y* axis. As to split-ring cavity, transverse (*x* axis) and longitudinal (*y* axis) polarized plane wave are simulated separately.

To obtain the resonance information for the two structures, we set a downward polarized plane wave incident vertical to the Si substrate. For the ring structure, the polarization direction is set as the *y*-axis. As to the split-ring cavity, transverse (*x*-axis) and longitudinal (*y*-axis) polarized plane waves are simulated separately.

**Figure 3:**
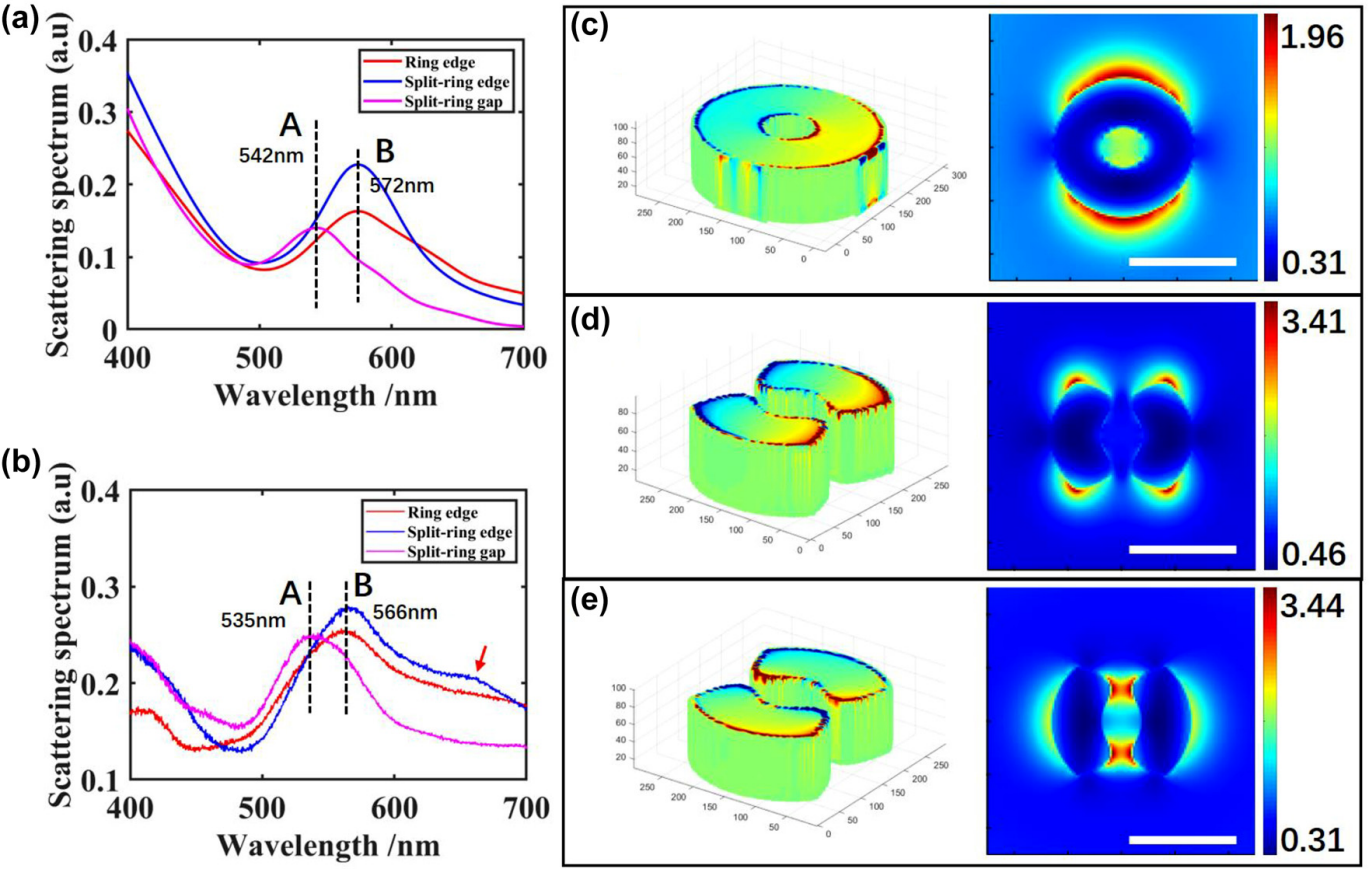
Scattering spectrums of plasmonic modes for (a) simulation and (b) experiment. Surface charge distribution and electric field distribution for (c) ring edge mode, (d) split-ring edge mode, and (e) split-ring gap mode. Scale bar: 200 nm.

As shown in Figure 3(a), the red line represents the simulated scattering spectrum for the ring cavity. One peak appears on 572 nm, which means a charge resonant mode. On the left part of Figure 3(c), surface charge distribution is calculated under the resonant wavelength. An electron dipole mode occurs on the two top corners on the outside surface of the ring. A 2D monitor is set on the cross-section at the middle height of the ring cavity to measure the energy distribution. As in the right part of Figure 3(c), under this dipole mode, most energy is localized on two outer edge of the ring structure. Thus, we can also name it as an edge mode. Here, we utilize a complementary harmonic inversion analysis method to simulate the quality factor (Q-factor) of resonances, which employs the filter diagonalization method to extract decay patterns in time series data [35, 36]. By this method, we can avoid simply evaluating the scattering spectrum or Fourier Transform of the temporal recorded signal from FDTD, by which we always underestimate the real value of the Q-factor. The time monitor is placed at the center of the top surface of the ring cavity, i.e., 80 nm height, and records the y-component of the electrical field temporal signal. The Q-factor for the edge mode under resonant wavelength is calculated as 8.48.

The same edge mode appears for the split-ring structure. Figure 3(d) shows the simulation results of the split-ring under longitudinally polarized light. Along the longitudinal direction, surface charges accumulate at the four outer edges of the corners. We can also identify this mode in the scattering spectrum, according to the same resonant peak wavelength as the ring structure in Figure 3(a). Four hot spots are generated outside the two arms, close to the farthest corners. The Q-factor for the edge mode is 7.50, which is also a small value.

However, apart from the edge mode, another resonant peak exists at 542 nm when transversely polarized light is illuminated to the split-ring. Surface charges concentrate on each long side with contrary charge quantities, which could also be treated as a dipole mode. This mode holds a Q-factor of 7.71 under the resonant wavelength. However, this dipole mode is cut off by the gaps, where a physical block of electrons results in a localized resonance of the cavity in the gaps. From near-field electric field distribution in Figure 3(e), we can observe high energy localized in the split-ring cavity at the two gaps along *y*-axis. Due to the hot spots generated in the gaps, this dipole mode could be characterized as a gap mode.

Experimental results agree well with simulation results. In Figure 3(b), we can find the edge mode for the ring structure appears at the wavelength of 566 nm. The edge mode and the gap mode for split-ring cavity are indicated at 566 nm and 534 nm, respectively. A small peak wavelength displacement for these two modes might be caused by a setting mismatch between the simulation and experiment. TFST source could be treated as a spectrum difference value between two simulations, where one contains nanostructures lying on the substrate and another one only contains the substrate [37]. Therefore, a displacement may occur when the substrate shows a large change of the reflection spectrum around the resonant peak, and the influence from this mismatch could almost be neglected for structures with a high Q-factor or substrate with a flat reflection spectrum. In addition, a small peak appears at 660 nm on the experimental scattering spectrum for split-ring edge mode. This fluctuation is supposed to be caused by unevenness of top surface during fabrication. (See Supporting Information and Figure S5). However, this experimental scattering spectrum could still demonstrate the resonant wavelengths we need to use for far-field mode distribution imaging.

We choose the center wavelength of 560 nm and 540 nm to conduct indirect parameter calculation for split-ring and ring cavity, respectively, which are close to their resonant peaks for better images. Also, related simulations are conducted by calculating electric field with the same procedure of PIMI measurement in FDTD. A 2D monitor is set at a height of 120 nm above the top surfaces of the structures to avoid the strong near-field evanescent wave. Ten images are saved with the rotation of the source polarization angle at a step of 18°, and subsequent calculations are performed in MATLAB.

**Figure 4:**
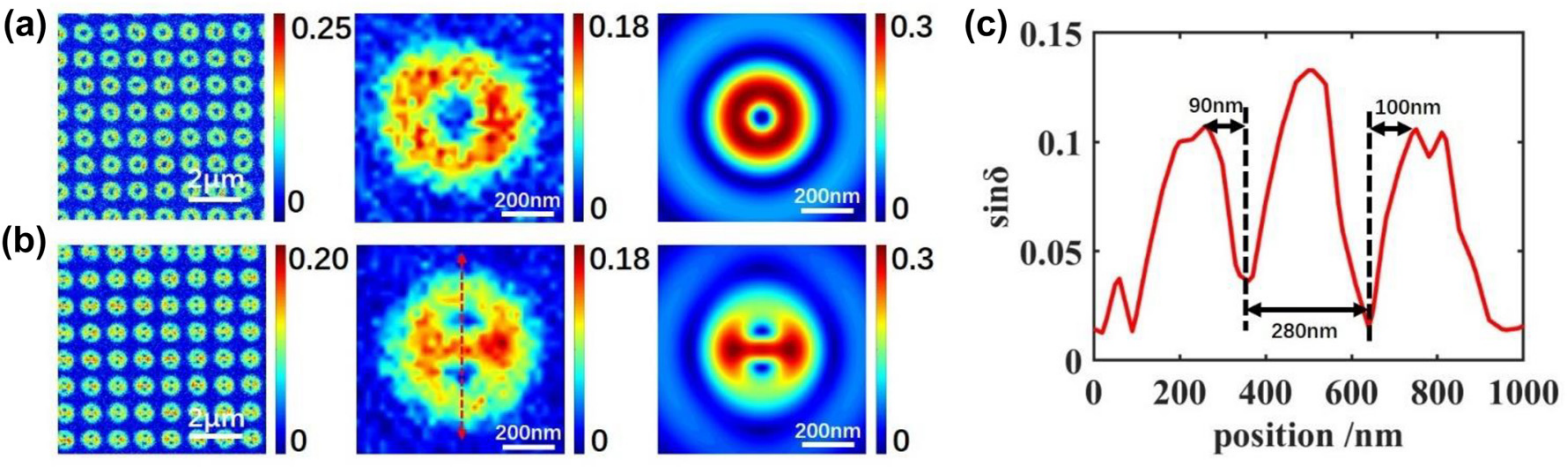
Sin *δ* images for (a) ring under 560 nm wavelength, and (b) split-ring under 540 nm wavelength. From left to right: large scale experimental results, single nanodot result, simulated single nanodot result. (c) Value of the spatial curve along red line in (b).

The experimental and simulated sin *δ* are shown in Figure 4. For ring structure, the sin *δ* result is also revealed to be a centrosymmetric shape, which is reasonable as edge modes could be excited equally under every direction. We also notice a singularity point occur at the center of the structure. This is because *E*_*t*_(*x*,*y*)^2^ and *E*_*l*_(*x*,*y*)^2^ in Eqs. (5) and (8) should be considered as the same edge modes with different orientations, which means the same value for *E*_*t*_(0,0)^2^ and *E*_*l*_(0,0)^2^ and results in a singularity zero value. The simulated sin *δ* of the ring structure shares the same shape as the experimental results.

Compared with the ring structure, the gap mode adds a contribution to the sin *δ* image of split-ring. As shown in both experimental and simulated results, two points of minimum value appear along the longitudinal symmetric axis with a small size. These two points should also be defined as singularity points. The relationship between these two points and modes is indicated below. From the formula of Δsin *δ*/sin *δ*, the singularity points occur only when *E*_*t*_(*x*,*y*)^2^ = *E*_*l*_(*x*,*y*)^2^, which is also the zero point for sin *δ*. As indicated in Figure 3d and e, hot spots from edge mode and gap mode in the split-ring are adjacent to each other, clamping the split-ring corners. Considering the Lagrange Middle-Value theorem, there must be a point that matches the condition of equality between two adjacent hot spots from different modes. Due to a very short distance from two adjacent hot spots, the singularity points could be considered as located near the top boundary corner of the split-ring. Therefore, four singularity points are generated symmetrically in four quadrants. However, when electromagnetic waves leave the surface of plasmonic structures, electric fields cannot hold their distribution as fine as in metal. As shown in Figure S6, two adjacent hot spots in the edge mode would merge as a larger flat hot spot in a short distance. Dispersion of electric hot spots will also lead to the mergences of two singularity points on two sides of the gap. As a consequence, the merged singularity points would form a flat shape, which is revealed in the experimental image, i.e., Figure 4(b). Simulated sin *δ* of split-ring at the near-field shows a similar pattern with two flat singularity points, which proves that the mergences of singularity points are near-field evolutions of electric field, instead of a far-field diffraction from the objective.

An intensity curve is shown in Figure 4(c) along red lines in Figure 4(b), to analyze the spatial characteristic of sin *δ*. In the curve, two valleys with sharp slop and peaks representing singularity points were shown, matching the theoretically sharp extremum in Eq. (8). The peak-valley widths for the two singularity points are measured as 90 nm and 100 nm, respectively, which means the resolving capability of the image could be down to 90 nm. This result is very comparable to the blur spot with a size of about 400 nm for split-ring from the conventional microscope under the diffraction limit of resolution. However, it is worthwhile to note that this cannot be directly defined as the spatial resolution due to the indirect relation between the singularity points and the nanostructure features.

The corresponding image and curve of Δsin *δ*/sin *δ* for the split-ring structure are also calculated in the Supporting Information and Figure S7. It should be noted that, the theoretical infinity values of Δsin *δ*/sin *δ* at the singularity points would always cover other spatial information. Also, the detection noise would be extremely large at some background sites where sin *δ* is small. Thus, sin *δ* is a suitable imaging parameter to display the structure features.

In addition, as mentioned before, singularity points are located within hot spots on the boundary corners. It means the singularity points could be utilized as a scale for far-field size estimation. As shown in Figure 4(c), the distance between two singularity points is 284 nm, which is very close to the split-ring size.

In fact, the features in sin *δ* images are strongly related to the structure details. In the Supporting Information, Figures S8 and S9 indicate simulation results when the size of rings changes or different directions of gaps are added. For a fixed outer radius *R*_out_ of the ring structure, the size of central singularity point in the sin *δ* image is constant with a similar value of *R*_out_. When the width of ring is fixed, the size of central singularity point will increase linearly. In addition, the sin *δ* parameter shows sensitivity to gaps on the ring structure. In Figure S9, singularity points at different directions appear at the position where gaps are added, which indicates a positioning ability of small nanogap structures.

To be noticed, Eq. (8) does not only affect singularity points in the sin *δ* images. At the boundary of the sin *δ* pattern, where sin *δ* nears zero, Δsin *δ*/sin *δ* could also be very large and generate a sharp peak. At boundaries in Figure 4(c), the sin *δ* values increase rapidly with a boundary width of about 110 nm.

**Figure 5:**
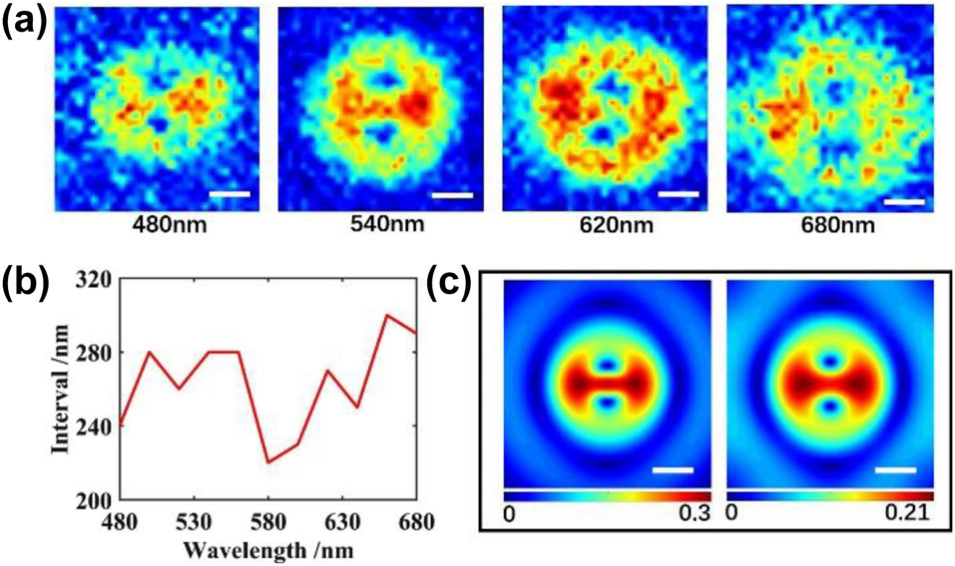
Influence caused by the change of wavelength and the diffraction. (a) Sin *δ* image of split-ring under 480 nm, 540 nm, 620 nm and 680 nm wavelength, (b) interval between two singularity points under different wavelengths, (c) simulated sin *δ* before and after diffraction. Scale bar: 200 nm.

Our detecting method also shows robustness when the imaging wavelength varies. Sin *δ* of a single split-ring under four different wavelengths are shown in Figure 5(a). We can observe relatively stable singularity points within a 200 nm wavelength range, and these characteristics could be detected even at wavelength far from the resonant wavelength.

With the illuminated wavelength increasing, the sin *δ* pattern will grow due to diffraction. However, the interval between two singularity points is stable from diffraction. As discussed above, the interval between singularity points under the 540 nm imaging wavelength is 280 nm, close to the split-ring size. Results under different wavelengths are shown in Figure 5(b) and no distinct variation of the interval appears in the curve. Another method is used to verify the diffraction resistance of the far-field mode characteristics in sin *δ*. Based on the simulated results under a wavelength of 540 nm, with an objective NA of 0.9, an airy disk operator with a diameter of 732 nm is convoluted into 10 raw images. A comparison in Figure 5(c) shows that the mode characteristics of the split-ring will not be heavily impacted by diffraction of the objective.

## 5 Conclusions

In conclusion, we have demonstrated a far-field method based on PIMI system to visualize plasmonic modes. From our derivation, singularity points would occur in the calculated PIMI sin *δ* image, which possesses a local high contrast, theoretically unlimited by the diffraction.

Two plasmonic structures are fabricated and imaged under PIMI system to verify our theoretical model. For ring structures, an edge mode is detected at 566 nm wavelength with energy concentrated on the outer surface of the structure, and the same mode is found in a split-ring cavity. However, another mode occurs under 534 nm with a gap surface charge distribution. This mode generates hot spots localized in the two corner sides of the split-ring gap.

Compared with the blur spot from a conventional microscope, sin *δ* image of ring and split-ring show much more spatial details related to their near-field modes. Sin *δ* of ring also reveals a centrosymmetric ring shape with a singularity point in the center. Two singularity points are generated in sin *δ* image of the split-ring. These points are associated with near-field hot spots for edge and gap modes.

Our system is capable of resolving spatial characteristics of plasmonic modes under 100 nm with high robustness to the wavelength variety and the diffraction in optical systems, which makes it suitable for high spatial-resolution characterization of subwavelength plasmonic structures with different resonance wavelengths. Due to the advantage of the far-field imaging process with a relatively large field of view, the system could be used for rapid large-scale plasmonic nanodevice characterization.

## Supplementary Material

Theory and calculation of PIMI; Diagram of PIMI system and the imaging process of split-ring under 540 nm illumination; Fabrication process of gold nanodot array; Fluctuation of scattering spectrum caused by uneven surfaces; The mergence of hot spots on two sides of the gap; Images and characteristic curve of sin δ and △sin δ/sin δ for split-ring; Relationship between sin δ features and gaps on thenanostructures.

## Supplementary Material

Supplementary Material Details
