# Supplementary material for: Sub-wavelength visualization of near-field scattering mode of plasmonic nano-cavity in the far-field
Source: Nanophotonics. 2023 Jan 16;12(2):297–305. doi: 10.1515/nanoph-2022-0679 (PMC11501192; doi:10.1515/nanoph-2022-0679)
Supplement: Supplementary file 1 — Supplementary Material Details [file j_nanoph-2022-0679_suppl_001.docx]

**Supporting information**

**Sub-wavelength Visualization of Near-Field Scattering Mode of Plasmonic Nano-Cavity in the Far-Field**

Xiao Jin^1,2^, Shengwei Ye^2^, Weiqing Cheng^2^, Jamie Jiangmin Hou^3^, Wanzhen Jin^1^, Tianyao Sheng^1^, Lianping Hou^2^, John H. Marsh^2^, Yefeng Yu^1^, Ming Sun^1^, Bin Ni^1^, Xuefeng Liu*^,1^ and Jichuan Xiong*^,1^

^1^School of Electronic and Optical Engineering, Nanjing University of Science and Technology, Nanjing 210094, P. R. China

^2^James Watt School of Engineering, University of Glasgow, Glasgow, G12 8QQ, UK

^3^The Royal College of Surgeons of Edinburgh, Nicolson Street, Edinburgh, Scotland, UK EH8 9DW

***Corresponding author:** [jichuan.xiong@njust.edu.cn](mailto:jichuan.xiong@njust.edu.cn) and liuxf_1956@sina.com

**This file includes:**

1. Theory and calculation of PIMI
2. Diagram of PIMI system and the imaging process of split-ring under 540 nm illumination
3. Fabrication process of gold nanodot array
4. Fluctuation of scattering spectrum caused by uneven surfaces
5. The mergence of hot spots on two sides of the gap
6. Images and characteristic curve of sinδ and Δsinδ/sinδ for split-ring
7. Relationship between sinδ features and gaps on the nanostructures

1. **Theory and calculation of PIMI**

PIMI system [1] is a method to observe birefringence in samples and image indirect parameters at a large scale. Considering a birefringent position on the sample, the phase difference between fast axis and slow axis is

$\delta=2\pi L\Delta n\text{/}\lambda$. (S1)

Here L is light path and λ is wavelength, and the polarization ellipse orientation angle $\phi$ is defined as angle between fast axis and X axis. In PIMI system, a rotating polarizer with angle of $\theta_{i}$ is placed before the sample, with reflected light from the sample sequncingly go through a quarter wave plate and a 45˚ polarizer. The output electric field and Intensity are


 (S2)

Where $I_{0}$ is the intensity under unpolarized illumination. And the PIMI results can be obtained by the following measurement procedure: the polarization angle $\theta_{i}$ of the illumination was modulated precisely from 0° through 180° in steps of 18°. By expanding Eq. (S2) trigonometrically, it can be reformulated in the following form:

$I_{i}=a_{0}+a_{1}sin2\theta_{i}+a_{2}\text{cos}2\theta_{i}$. (S3)

. (S4)

With a total number of steps N = 180°/18°, $a_{0}$, $a_{1}$ and $a_{2}$ can be calculated as:

$a_{0}=\sum_{i=1}^{N} \frac{1}{N}I_{i},a_{1}=\sum_{i=1}^{N} \frac{2}{N}I_{i}sin\alpha_{i},a_{2}=\sum_{i=1}^{N} \frac{2}{N}I_{i}cos\alpha_{i}$ (S5)

Thus, the PIMI parameters, $sin\delta$ and $\phi$ can be extracted by utilizing the above equations.

 (S6)

It should be noted that, the system was initially designed to detect birefringence in samples. However, For plasmonic structures, the character parameter sinδ could still be calculated as a different indirect parameter to reveal plasmonic responses of polarization. We utilize the same detection method and parameter name for the new theory in the manuscript.

[1] Liu X, Qiu B, Chen Q, et al. Characterization of graphene layers using super resolution polarization parameter indirect microscopic imaging[J]. Optics express, 2014, 22(17): 20446-20456.

1. **Diagram of PIMI system and the imaging process of split-ring under 540 nm illumination**


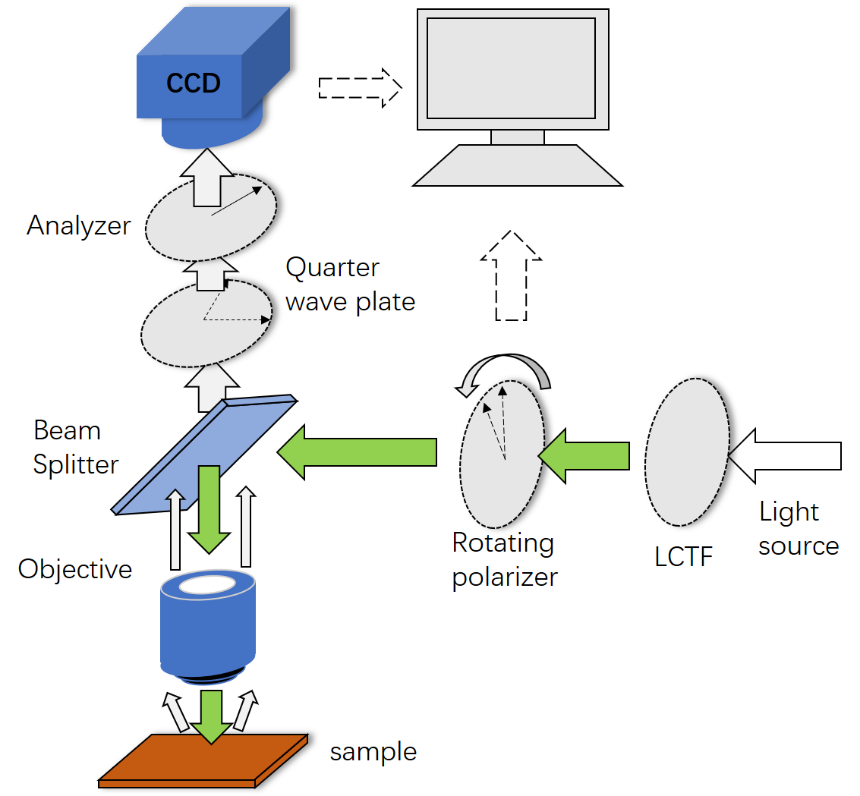


Fig. S1. Schematic diagram of PIMI system


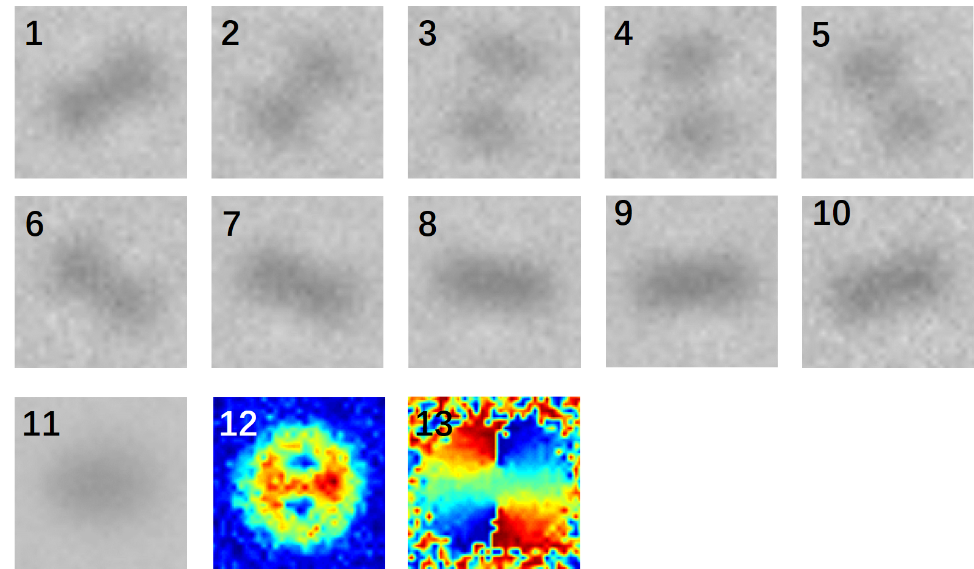


Fig. S2. 1-10: 10 PIMI raw images of simulation under a rotating illumination polarization with a step of 18°, 11: image of $I_{0}$, 12: PIMI sinδ image, 13: PIMI ϕ image.


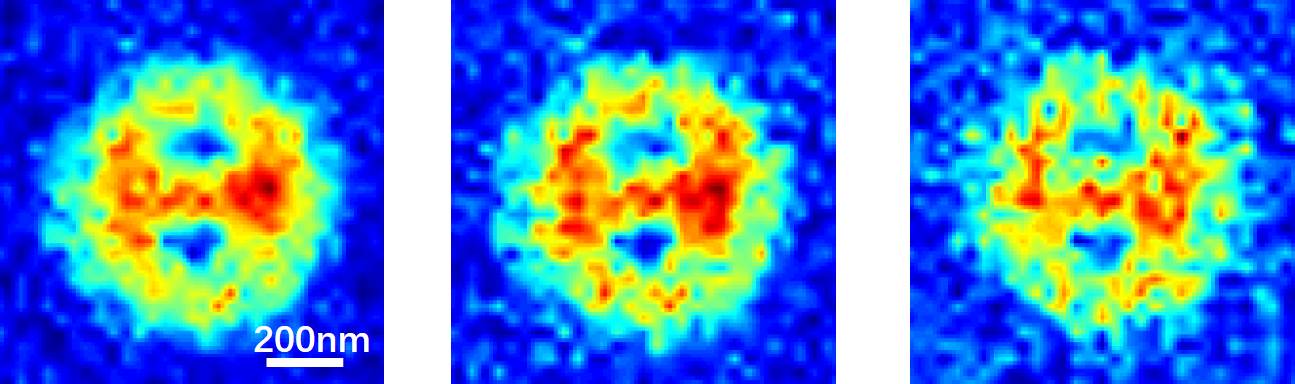


Fig. S3. sinδ images calculated from 10, 8 and 5 angles (from left to right).

Theoretically in PIMI method, any numbers of images from four to infinity could be used for calculating. The number of images actually represents the sampling number on an intensity variation curve with respect to the rotating polarization angle, in order to perform a Fourier calculation and filtering. Theoretically, the polarization sampling should cover at least a half period of the variation curve and a more sampling number leads to more accurately calculated PIMI images. However, a measurement for many angles is time-consuming and may subject to a possible platform drifting of the microscopic system. Therefore, the measurements should be completed as soon as possible, which limits the number of polarization sampling. Here, we give sinδ images calculated with different numbers of polarization samplings in Fig S3. Obviously, a small number of measurements leads lower signal-to-noise ratio due to the inevitable systematic errors and random noises. Thus, 10 angles is a compromised number to make a balance between the image quality and a reasonable measurement time within about 20 s.

1. **The fabrication process of gold nanodot array**

The array of plasmonic array was realized by following fabrication processes. A silicon sample was first cleaned by acetone and Isopropyl Alcohol (IPA) solvents separately. These two steps were carried out in a water bath at a temperature of 50 °C. Then, ultrasonic cleaning was applied for better cleaning effects at room temperature. After that, a 200 nm thick layer Polymethyl Methacrylate (PMMA) photoresist was spun on the surface of the silicon sample by controlling the spinning speed.

Electron beam lithography (EBL) was used to define the patterns on the PMMA resist with a high resolution. After development, the gold nanodot array pattern was transferred to PMMA. Next, a 10 nm thick titanium layer and a 70 nm thick gold layer were deposited successively on the surface of the silicon sample by the Electron Beam Evaporation method. The 10 nm thick titanium was first deposited mainly to enhance the metal adhesion with the silicon surface. After a standard lift-off process, the gold nanodot array was finally realized.


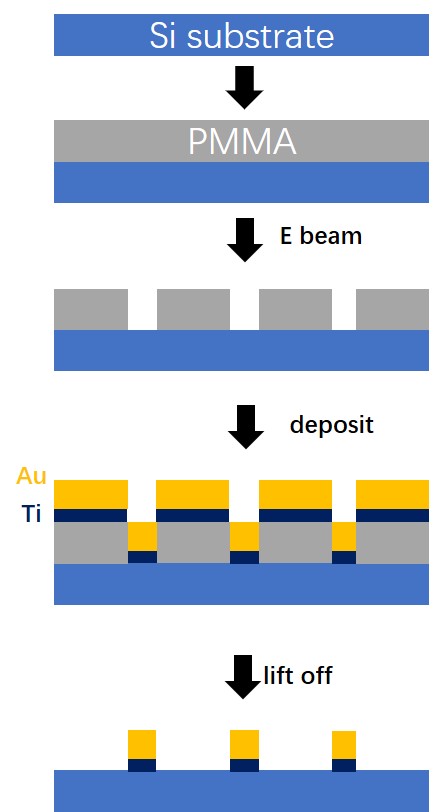


Fig. S4 Fabrication processes of gold nanodot array

1. **Fluctuation of scattering spectrum caused by uneven surfaces**

We think the peak at about 660 nm for the split-ring edge mode is due to the unevenness of the top surface. As shown in Fig. 8(a), during the fabrication process, the Au layer will not be deposited uniformly near the boundary of PMMA. As a result, the thickness of Au at the edge of the structure is higher than that at the central part. In SEM results, the central part of the two splitting arms is darker than the edge, i.e., the red arrow in the SEM image of Fig. 8(a), agrees with the height difference caused by the fabrication.


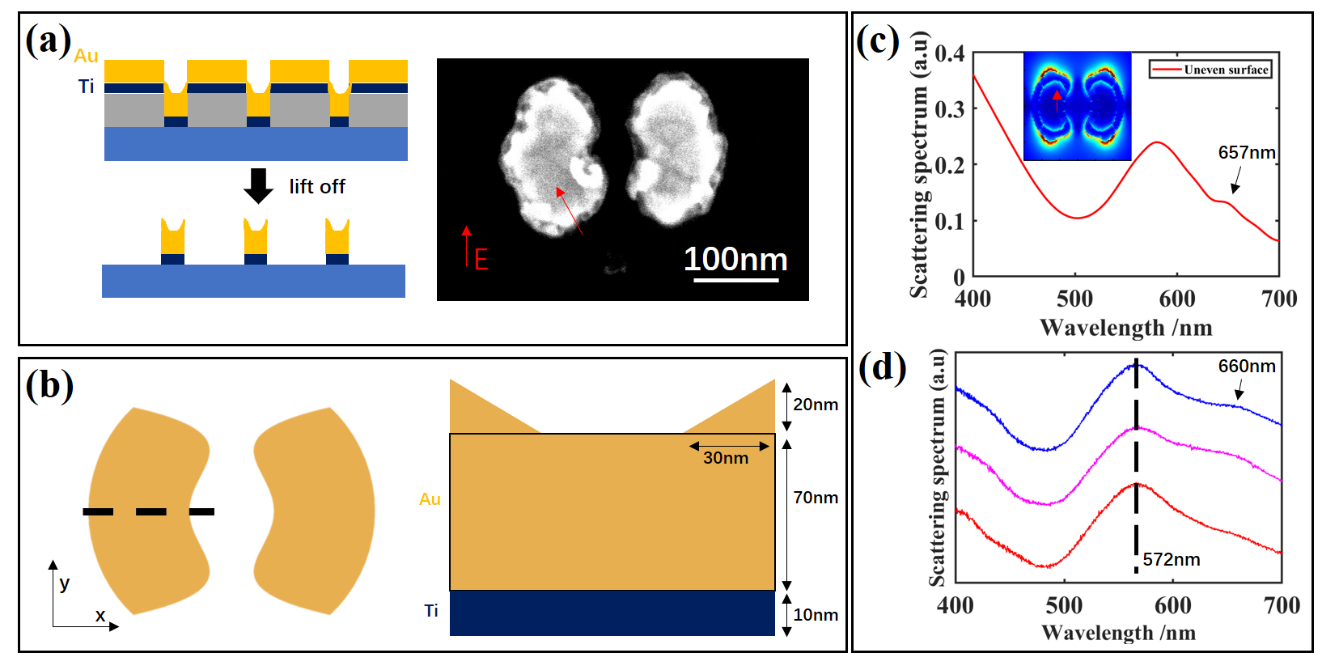


Fig. S5. (a) The fabrication process and the SEM image of the split-ring with an uneven surface. (b) the configuration of the uneven surface. (c) The simulated scattering spectrum for the uneven surface in (b) under Y polarization and corresponding electric field on the height of 80 nm. (d) Experimental scattering spectrum at different sites under Y polarization.

To verify the influence of the uneven surface, the height is set with a gradient decrease at the edges of the split-ring structures in the simulation for Fig. 8(c). The cross-section of one arm of the split-ring is shown in Fig. 8(b), where a bonus height of 20 nm and a width of 30 nm are added to the edge of the ring to create an uneven surface in the simulation. The simulation results for the uneven surfaces under the Y polarization illumination are shown in Fig.8(c). A small peak exists at 657 nm on the scattering spectrum, which is near the peak of 660 nm in the experiment results. Under this wavelength, the electric field on the top surface could characterize the impact from the uneven surfaces.

As labeled by the black arrow, a weak localized energy occurs which indicates an inner resonance generated by the rising edges of the splitting arms. However, this inner mode is too weak, compared with the gap mode or the edge mode. In addition, the features of the uneven surfaces could not be constructed stably during the fabrication, leading to an unstable generation of the inner mode. We choose three different sites on the nano array and plot their scattering spectrums in Fig. 8(d). The strong longitudinal dipole mode at 572 nm is stable for all curves. Nevertheless, the weak mode generated by the uneven surface sometimes disappears as a result of fabrication errors. In future work, the necessary polishing process would be applied to achieve a smooth top surface and reduce unexpected modes.

1. **The mergence of hot spots** **on two sides of the gap**

From the discussion in the manuscript, a singularity point will exist at a position between two hot spots in the edge mode and the gap mode. Thus, the number of singularity points is determined by the number of electric field hot spots. However, the localized energy cannot be constrained any more when the scattering field leaves the metal surface when propagating to the far field.


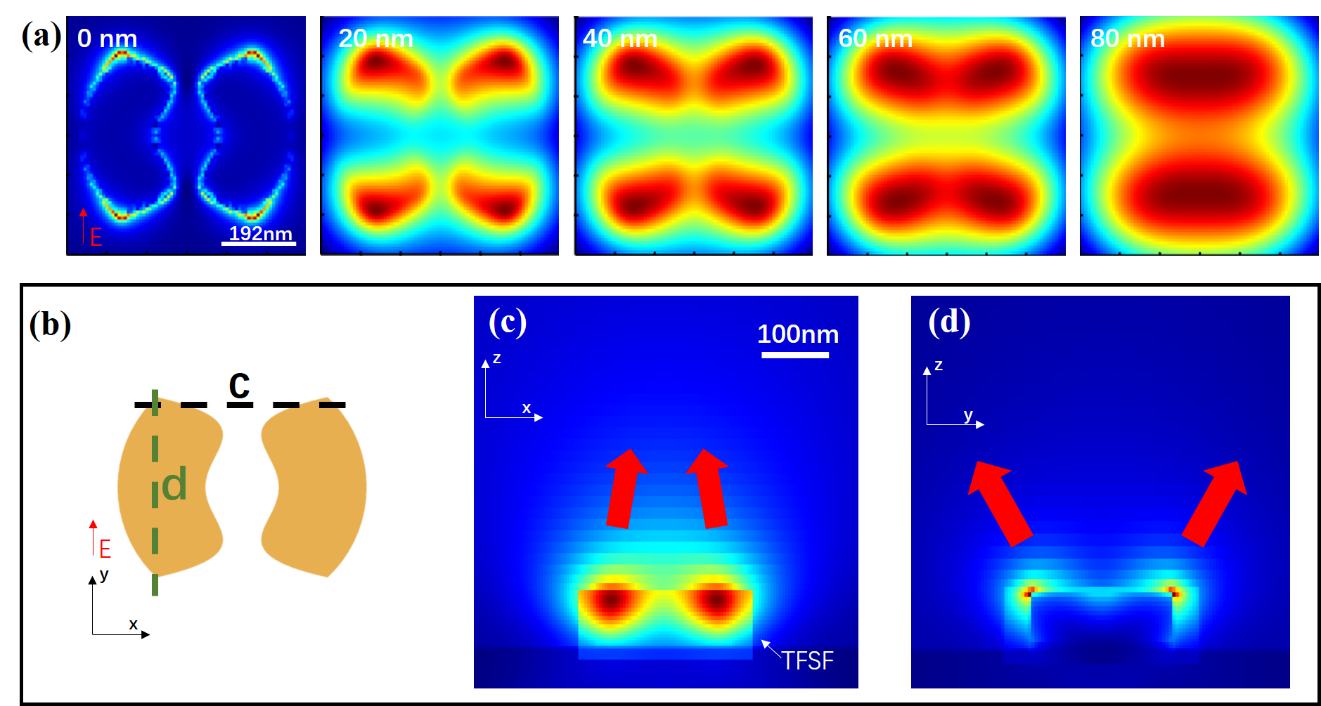


Fig. S6. (a) Electric field distribution at different heights above the top surface. (b) positions for two cross sections shown in (c) and (d). (c) x-z cross section of the electric field along the black line in (b). (d) y-z cross section of the electric field along the black line in (b).

In Fig. S6 (a), we set a series of monitors at different heights above the top surface of split-ring structures under an incident light with Y polarization. The two upper localized hot spots start to merge after the monitor leaves the structure surface, and form as a larger single hot spot rapidly when the scattering field propagates to a 80 nm height.

It is clearer to investigate the mergences of hot spots along X-Z and Y-Z cross sections. In Fig. S6 (c), two upper hot spots propagate along the Z direction and naturally mix to a single part. Nevertheless, we could find a tendency of divergency for two hot spots along the Y direction, preventing them merge into one, as shown in Fig. S6(d). This divergency at symmetric edges with opposite phases of electrons is common for plasmonic structures on a substrate, as a result of the hybridization of the scattering electromagnetic field and the electromagnetic field reflected from the substrate surface [2]. As to hot spots in Fig. S6(c), they are originally one continuous hot spot generated by one side of longitudinal dipole mode with the same phase of electrons in a completed ring structure. And this hot spot was split into two by the physical cut-off of the ring at the opening gap. Thus, the electric fields from the split hot spots show a tendency of merging when propagating in the Z direction, contrary to the tendency in Fig. S6(d). In the manuscript, the calculated sinδ parameter image is reflecting this. The mergences of electric field hot spots by the mergences of singularity points in the imaging results.

[2] Ding S Y, Yi J, Li J F, et al. Nanostructure-based plasmon-enhanced Raman spectroscopy for surface analysis of materials[J]. Nature Reviews Materials, 2016, 1(6): 1-16.

1. **Images and characteristic curves of sinδ and Δsinδ/sinδ for split-ring**


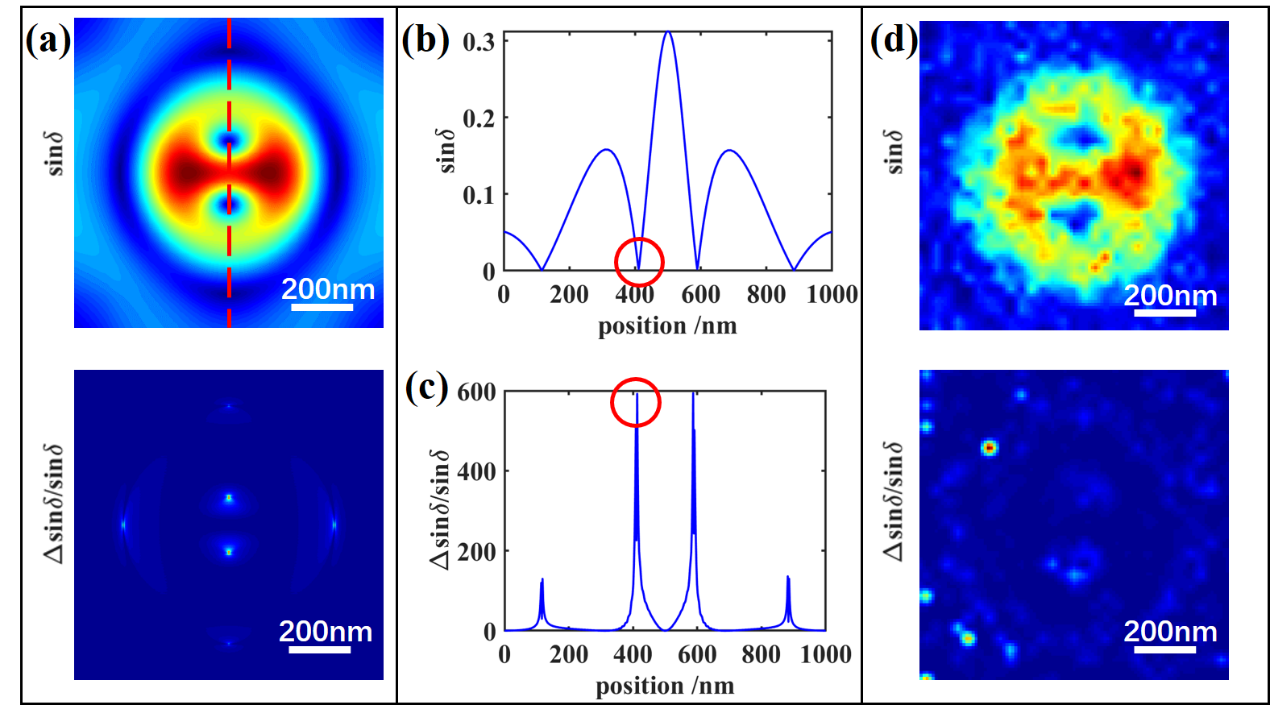


Fig. S7. (a) simulated sinδ and Δsinδ/sinδ image for the split-ring structure under the wavelength of 540 nm. (b) sinδ and (c) Δsinδ/sinδ curves along the red line shown in (a). (d) experimental sinδ and Δsinδ/sinδ image for the split-ring structure under the wavelength of 540 nm.

In the simulation, the extremely high value of Δsinδ/sinδ at singularity points would cover other spatial distribution information of Δsinδ/sinδ with low values, although two sharp spots indicating the singularity points can be identified easily, the second row of Fig. S7(a). While the sinδ image can indicate the singularity points with zero-value points and retain the spatial distribution information in the meantime. Both in simulation and experiment results, the singularity points can also be identified from the sinδ image, without losing the spatial distribution information. On contrary, in the second row of Fig. S7(d), the Δsinδ/sinδ image shows a lower signal-to-noise ratio due to the imaging noise and systematic errors. Only two blurred bright spots were shown at the expected positions of the two singularity points. Therefore, we believe that sinδ could be a more suitable parameter to be used to reveal the singularity points together with the mode distributions.

1. **Relationship between sinδ features and the configuration of nanostructures**

To clarify the relationship between the size of ring structures and sinδ features, two series of ring structures are simulated under the wavelength of 540 nm and shown in Fig S8. Other structure parameters and the simulation configuration are consistence with the manuscript. To control variables, we did not change the illumination wavelength for every structure.

In Fig S8(a), we set the outer radius of the ring structure $R_{out}$ as a fixed value of 130 nm, and the inner radius $R_{in}$ varies from 0 nm to 100 nm. The sinδ curves are drawn along the red line in the sinδ image. With the size of the central hole of the ring increases, the values of sinδ decrease rapidly. However, the curve shapes remain a similar distribution as shown in Fig. S8(a). We also notice a growing intensity ratio of another ring in sinδ, which might indicate a stronger high order interference between the scattering light and substrate reflection when these two fields reach to similar intensity levels [3, 4]. The interval between the first two maximum points starting from the center, i.e., the radius of the central ring of the sinδ image, is marked with a red circle in Fig. S8(a) and plotted with respect to $R_{in}$ in Fig S8(b). The radius of the central ring is stable with a value of about 135 nm, which is almost the size of $R_{out}$.


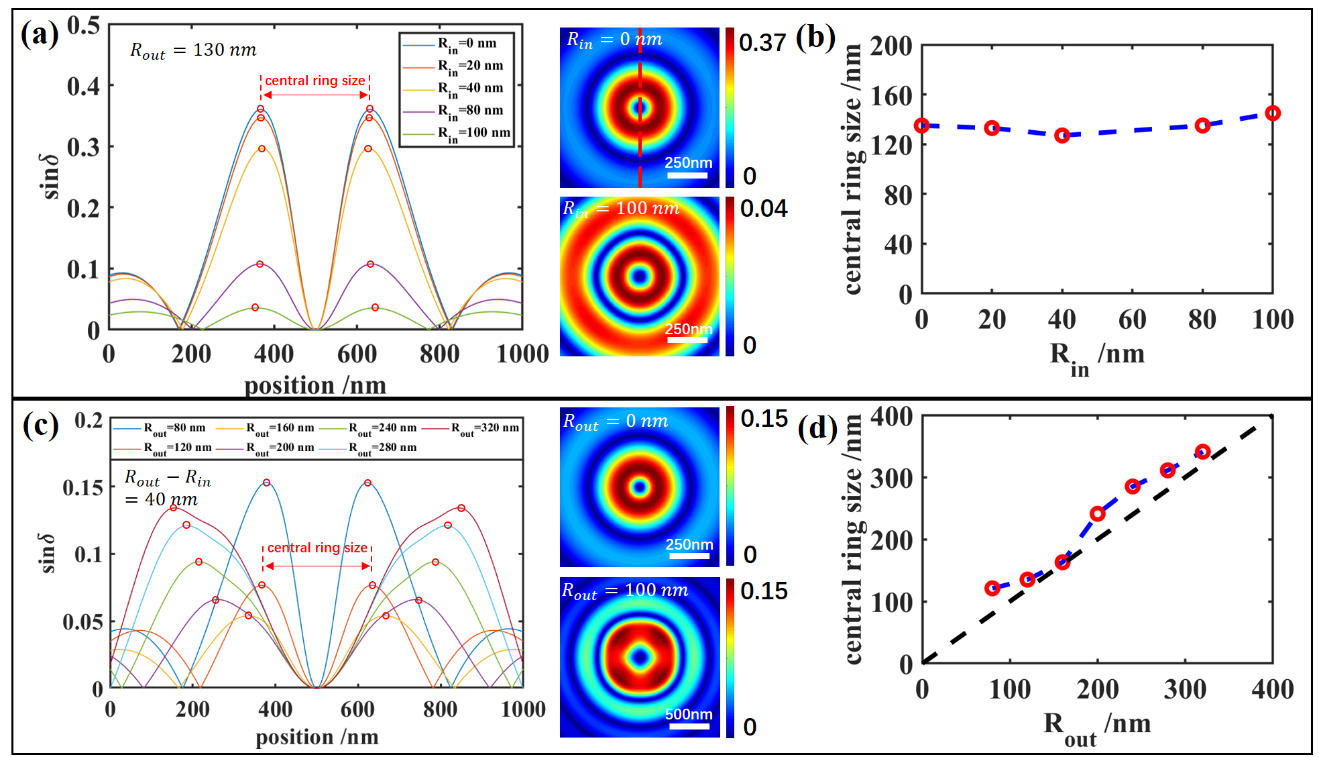


Fig. S8. (a) sinδ curves and images for ring structures with different inner radiuses. (b) the size of the central ring of the sinδ image, with maximum marked by red circles in (a), with respect to the variation of central hole sizes of the structure. (c) sinδ curves and images for ring structures with different outer radiuses. (d) the size of the central ring of the sinδ image, with maximum marked by red circles in (c), with respect to the variation of the outer radius of the structure.

Then we fix the width of Au ring $R_{out}-R_{in}$ to 40 nm, raising $R_{out}$ and calculate analogous curves in Fig S8(c) and (d). Different from results for a fixed $R_{out}$, the central ring size in sinδ shows a high linearity with the size of $R_{out}$ when $R_{out}$ increases, as shown in Fig S8(d), with a value slightly larger than their corresponding $R_{out}$. It could be concluded that, the inner radius of the ring structure show influences more to the overall magnitude of sinδ, and the central ring size of the sinδ image are highly dependent on the outer radius of the ring structure. This phenomenon is reasonable for a localized surface plasmonic structure, where resonance always happened at the edges or outer surfaces and causes corresponding scattering changes at these positions.

As to the different positions of gaps, a modified split-ring structure with an outer radius of 200 nm, an inner radius of 150 nm and 10° opening angle of gaps is simulated to clarify the influences caused by the positions of gaps. The size is different from which in the manuscript, where the opening angle of the gap used in the article is too large, leading to an overlap of gaps along X and Y axes. The results are shown in Fig S9.


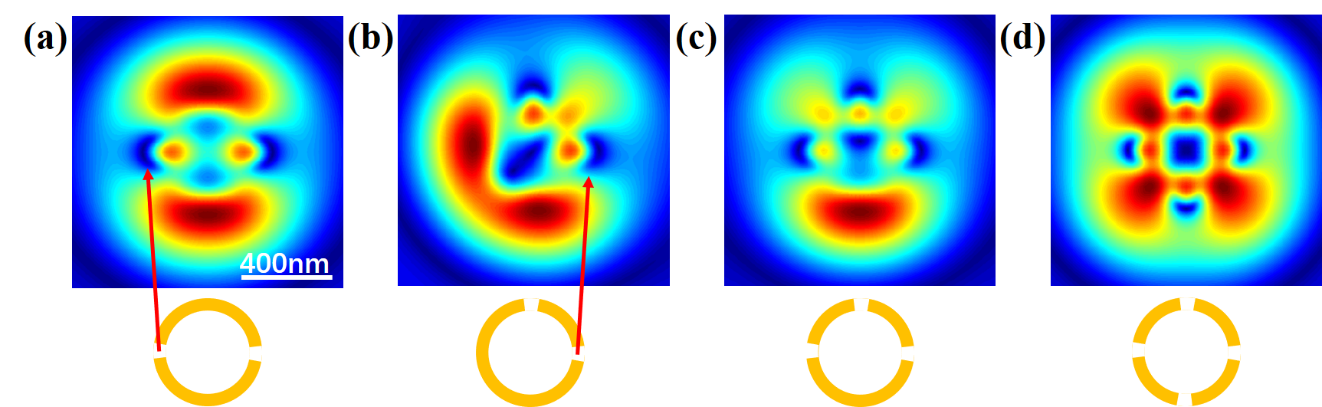


Fig. S9. Split-ring structures with different directions of gaps and their sinδ results

When the split positions are along the X-axis, the singularity points appear at X-axis, exactly the positions of gaps. The asymmetric results in Fig S9(b) and (c) could also clearly identify the positions of gaps by singularity points, which proves a strong connection between singularity points and gaps. To be noticed, a fundamental central singularity point for the basic ring structure is generated at the center of the image. In the manuscript, this central singularity point for the basic ring does not exist for a reason that two opposite gaps are relatively too large and the basic ring shape is highly broken.

[3] Pin-Tian Lyu, Qing-Yue Li, Pei Wu, Chao Sun, Bin Kang, Hong-Yuan Chen, and Jing-Juan Xu. Decrypting Material Performance by Wide-field Femtosecond Interferometric Imaging of Energy Carrier Evolution[J]. Journal of the American Chemical Society. 2022 144 (30), 13928-13937.

[4] Lin S, He Y, Feng D, et al. Optical Fingerprint of Flat Substrate Surface and Marker-Free Lateral Displacement Detection with Angstrom-Level Precision[J]. Physical Review Letters, 2022, 129(21): 213201.
